# Supplementary material for: Exploring Biomolecular Self-Assembly with Far-Infrared Radiation
Source: Biomolecules. 2022 Sep 19;12(9):1326. doi: 10.3390/biom12091326 (PMC9496551; doi:10.3390/biom12091326)
Supplement: Supplementary file 1 [file biomolecules-12-01326-s001.zip › biomolecules-1903728-supplementary.pdf]

biomolecules

MDPI

Review

Exploring Biomolecular Self-Assembly with Far-Infrared Radiation

Takayasu Kawasaki [1,*], Yuusuke Yamaguchi [2], Hideaki Kitahara [2], Akinori Irizawa [3] and Masahiko Tani [2]

[1] Accelerator Laboratory, High Energy Accelerator Research Organization, 1-1 Oho, Tsukuba, Ibaraki 305-0801, Japan
[2] Research Center for Development of Far-Infrared Region, University of Fukui, 3-9-1 Bunkyo, Fukui, Fukui 910-8507, Japan, y-yama@fir.u-fukui.ac.jp; kitahara@fir.u-fukui.ac.jp; tani@fir.u-fukui.ac.jp
[3] SR Center, Research Organization of Science and Technology, Ritsumeikan University, 1-1-1 Nojihigashi, Kusatsu, Shiga 525-8577, Japan, irizawa@fc.ritsumei.ac.jp
* Correspondence: takayasu.kawasaki@kek.jp

Abstract: Physical engineering technology using far-infrared radiation has been gathering attention in chemical, biological, and material research fields. In particular, the high-power radiation at the terahertz region can give remarkable effects on biological materials distinct from a simple thermal treatment. Self-assembly of biological molecules such as amyloid proteins and cellulose fiber plays various roles in medical and biomaterials fields. A common characteristic of those biomolecular aggregates is a sheet-like fibrous structure that is rigid and insoluble in water, and it is often hard to manipulate the stacking conformation without heating, organic solvents, or chemical reagents. We discovered that those fibrous formats can be conformationally regulated by means of intense far-infrared radiations from a free-electron laser and gyrotron. In this review, we would like to show the latest and the past studies on the effects of far-infrared radiation on the fibrous biomaterials and to suggest the potential use of the far-infrared radiation for regulation of the biomolecular self-assembly.

Keywords: terahertz; far-infrared radiation; amyloid; cellulose; free-electron laser; gyrotron; self-assembly

Citation: Kawasaki, T.; Yamaguchi, Y.; Kitahara, H.; Irizawa, A.; Tani, M. Exploring Biomolecular Self-Assembly with Far-infrared Radiation. *Biomolecules* **2022**, *12*, 1326. https://doi.org/10.3390/biom12091326

Academic Editors: Anna Mitraki, Chrysoula Kokotidou, Vagelis Harmandaris and Anastassia N. Rissanou

Received: 23 August 2022
Accepted: 17 September 2022
Published: 19 September 2022

Publisher's Note: MDPI stays neutral with regard to jurisdictional claims in published maps and institutional affiliations.

Copyright: © 2022 by the authors. Licensee MDPI, Basel, Switzerland. This article is an open access article distributed under the terms and conditions of the Creative Commons Attribution (CC BY) license (https://creativecommons.org/licenses/by/4.0/).

1. Materials

Lysozyme from hen egg white (3 crystallized, > 9000 U mg^{-1} protein) and Congo-red were purchased from Sigma-Aldrich (Tokyo, Japan). Phosphate buffered saline (PBS, pH at 25 °C: 7.2–7.4), acetic acid, and sodium chloride were purchased from Wako Pure Chemical Industries (Osaka, Japan). Cellulose powder (fibers) was purchased from Merck Co (Tokyo, Japan).

2. Sample Preparation

For THz-FEL irradiation, lysozyme was fibrillated as follows: the powder was dissolved in 20 % acetic acid (2.5 mg mL^{-1}) containing sodium chloride (0.5 M), and the solution (1 mL) was incubated for 20 h at 37 °C. The resulting precipitate was collected by centrifugation (14000 rpm, 10 min) and suspended in water. The suspension (10 μL) was dropped on a stainless steel plate for infrared microspectroscopy or a glass slide for scanning-electron microscopy observation. After drying under atmospheric conditions, the sample was irradiated by the THz-FEL.

As for the gyrotron experiments, the lysozyme was dissolved in acidic water (150 μL) as described above, and the solution was directly used for the irradiation experiment without subsequent thermal incubation.

3. THz-FEL Irradiation

The principle of the beam generation is briefly described as follows: an FEL oscillation system consists of an electron gun, sub-harmonic buncher, an accelerator tube, a periodic magnetic field (wiggler in this case), and an optical cavity to amplify the FEL pulses (Fig. S1). A small portion of the FEL pulses in the cavity is extracted via a coupling hole that is 3 mm in diameter at the center of the upstream resonant mirror. The wiggler is a Halbach-type magnetic field. The FEL beam is transported through a concrete wall (3 m in thick) and through a diamond window of the monochromator to the experimental room. The sample dried on a slide base was irradiated by the THz-FEL from the vertical direction at room temperature with raster scan. Under the irradiation conditions, the beam diameter was focused to approximately 400 μm by using a parabolic reflector where the irradiation area was 1 mm x 1mm square, while the step scan length was set to 0.1 mm.

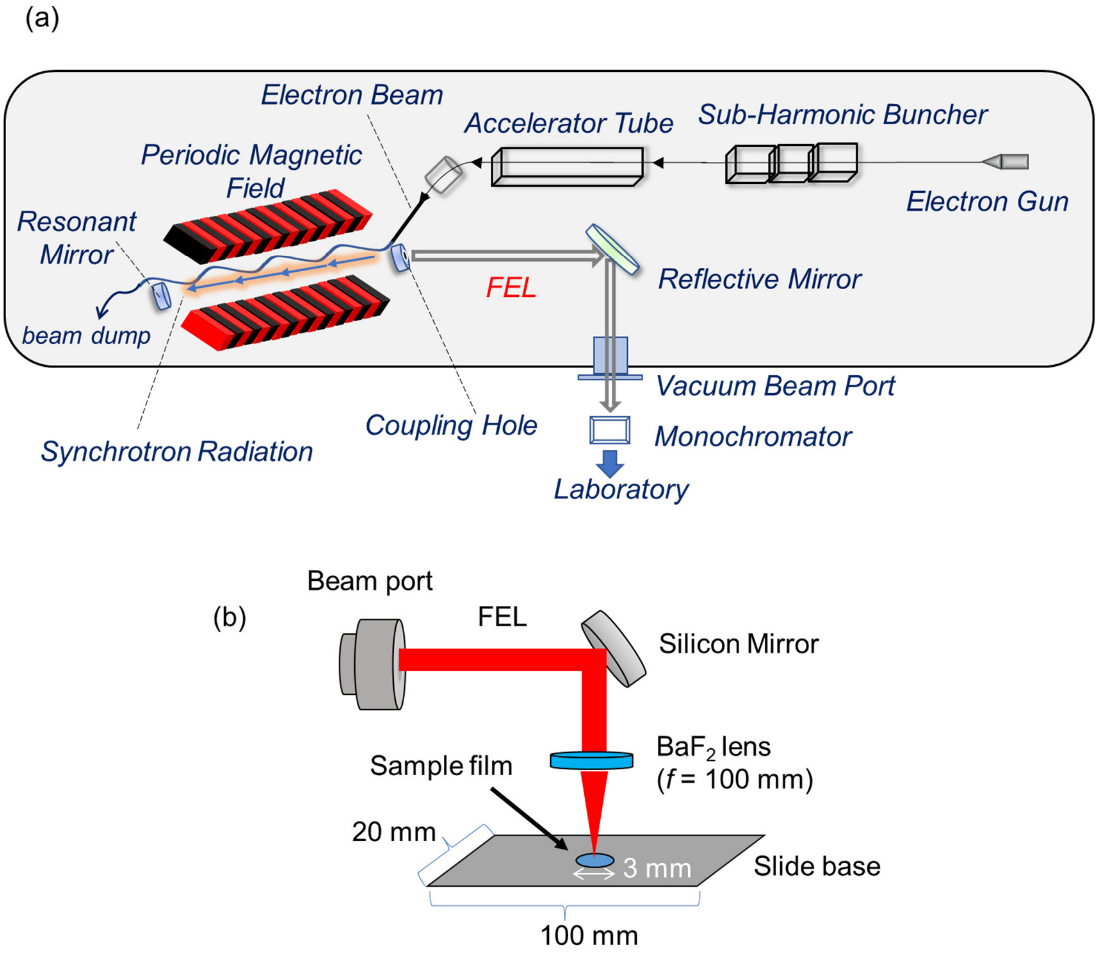

Figure S1. Oscillation system of THz-FEL and the irradiation setup. The system is composed of four parts (**a**): linear accelerator (Electron Gun + Sub-Harmonic Buncher + Accelerator Tube), Periodic Magnetic Field, Resonant Mirrors, and outlet of light (Reflective Mirror and Vacuum Beam Port). (**b**) Sample geometry. The beam direction was controlled using silicon mirror and focused onto the sample film on a slide base by using BaF_2 lens.

4. Gyrotron Irradiation System

The gyrotron is a vacuum electron tube where the operation is based on the phenomenon known as electron cyclotron maser instability (Fig. S2). The structure is composed of an electron-optical system based on a triode magnetron injection gun with a thermionic cathode that generates a helical electron beam in the superconducting magnet, a cavity resonator for coupling the electron beams with waves, an internal mode converter to

adjust spatial distributions of oscillated waves, an output vacuum window, and a water-cooled collector of the spent electron beams. The submillimeter wave is oscillated as a Gaussian wave beam from the output vacuum window. We used the Gyrotron FU CW GVIB far-infrared radiation system which can expose samples to a 420 GHz wave with 10 W power. The radiation wavelength was 720 μm, and the pulse duration was set to either 1 ms or 2 ms at 5 Hz repetition. The temperature increase of the sample during the irradiation was monitored using a Testo 875 thermography camera (Testo). The sample in aqueous solution (150 μL) was put on an Eppendorf tube that is made of polypropylene and was irradiated at ambient temperature (ca. 25 °C) from the vertical direction.

(a)

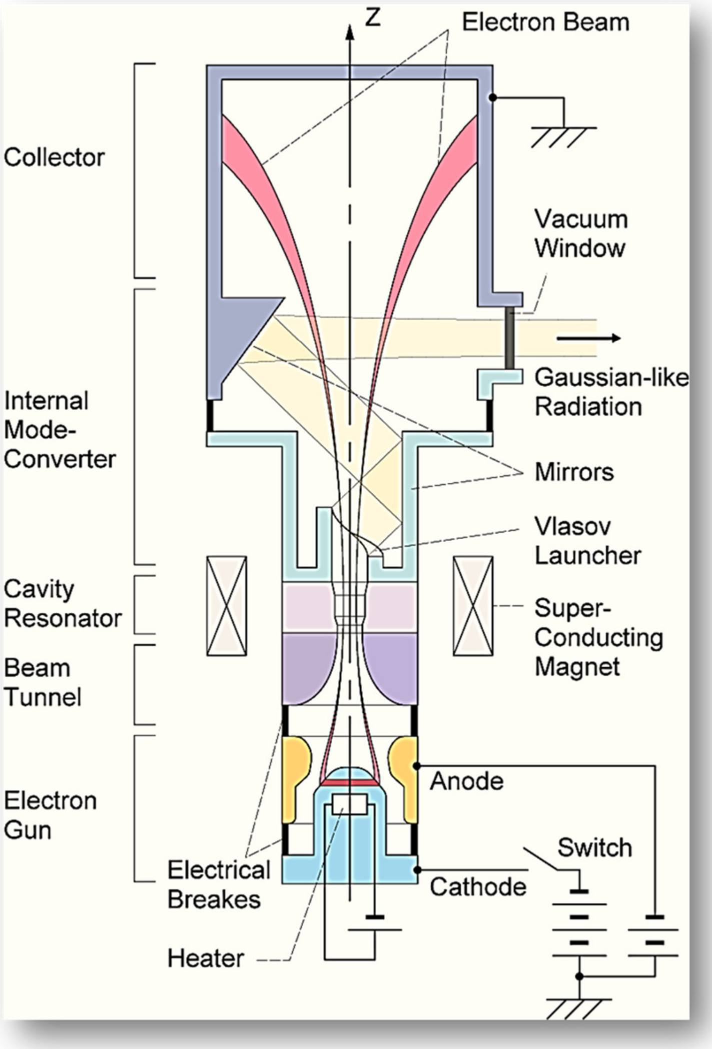

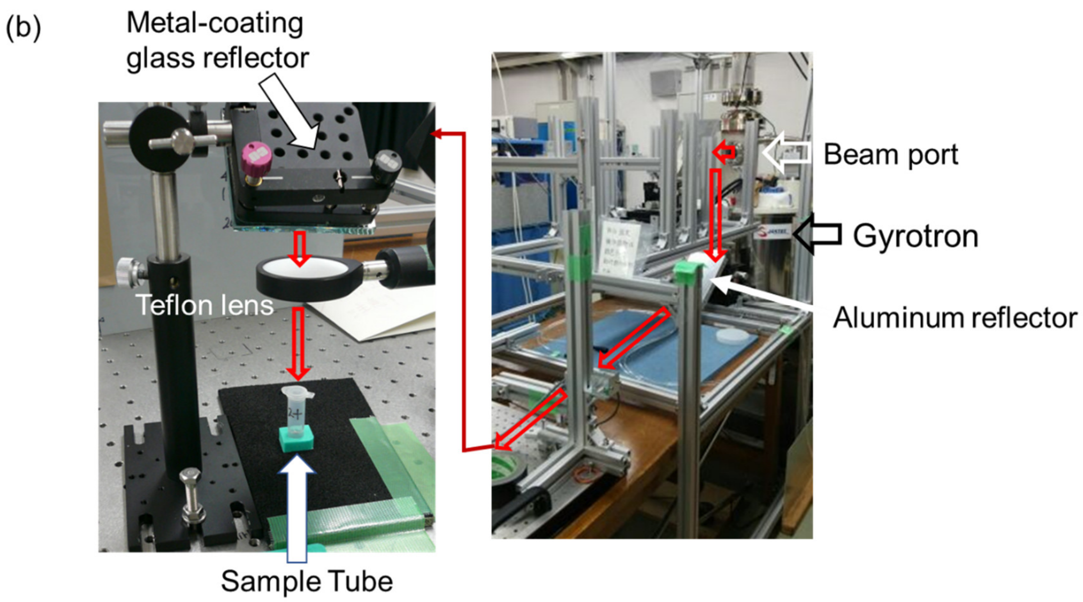

Figure S2. Oscillation system of gyrotron and the irradiation setup. The overall structure is briefly composed of five parts (**a**): Electron Gun, Beam Tunnel, Cavity Resonator, Internal Mode Converter, and Collector. The submillimeter wave is radiated from Vacuum Window. (**b**) Sample geometry. Beam line from the beam port to the sample was shown as red arrows. The beam direction is controlled by using aluminum reflectors and metal-coating glass mirror. The submillimeter wave was focused on the sample tube by using Teflon lens (f = 10 cm).

5. Measurement of Transmittance of Terahertz Wave against Plastic Tube

We employed THz-time-domain spectroscopy instrument at the Research Center for Development of Far-Infrared Region, University of Fukui. The sample tube that is composed of polypropylene polymer was processed on a hot plate to be extended as a thin sheet (1.4 mm in thickness) and mounted on a sample holder which was vertically positioned to the THz beam line. The erbium-doped fiber laser with a second-harmonic generation module was used to excite a photoconductive antenna, which emits THz radiation in free space. The laser light source emits a pulse of 150-fs width at a repetition rate of 40 MHz, and its fundamental central wavelength of 1.56 μm served as the optical pump source. The second harmonic output (about 20 mW) at 790 nm was used to pump the photoconductive antennas used as the emitter and detector of the THz pulsed radiation. The waveforms of the electric field of THz waves, with and without the sample, were measured by scanning the optical delay line. The THz waveform was acquired by means of 10 scans per observation in the experiment, and each sample was observed thrice. The averaged time-domain waveforms were Fourier transformed to the sample and the reference spectrum, from which the transmittance spectra were deduced.

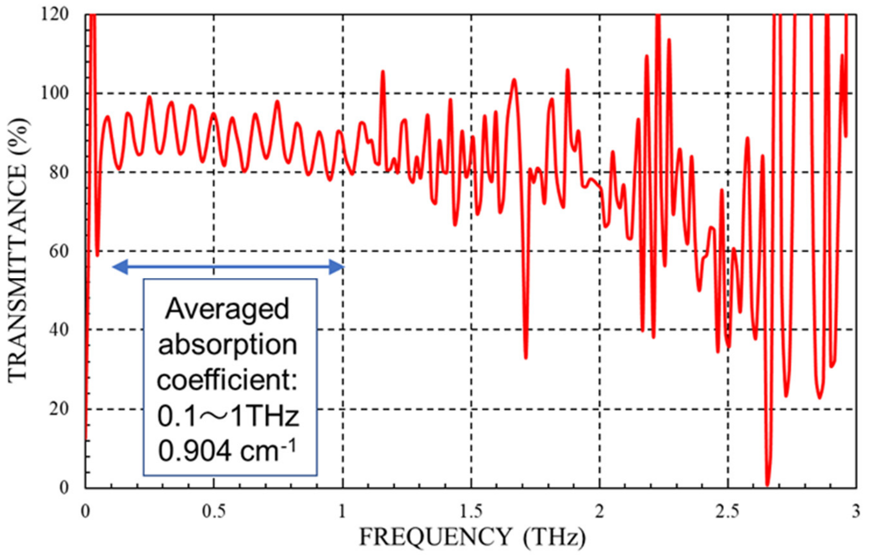

Figure S3. Transmittance of sample tube at 0–2.0 THz region.

6. Terahertz Spectroscopy

We used a far-infrared Fourier-transform spectrometer (IFS66v/S, Bruker) for the absorption spectrum measurement for lysozyme at the terahertz region. The sample powder was mixed with CsI powder and pressed to form a mini-disk plate. The measurement was performed by transmission mode, and the spectrum was recorded at 130–700 cm^{-1} with 32 scans using Mylar (polyester film) as beam splitter.

7. Infrared Microspectroscopy

The mid-IR spectra for lysozyme were measured using IRT-7000 infrared microscope (Jasco Co, Tokyo, Japan) and FT/IR-6100 spectrometer (Jasco Co., Tokyo, Japan). The dry surface of the sample film was observed by using a 16x Cassegrain lens, and the infrared spectra were recorded via a reflection mode with 64 scans and 4 cm^{-1} resolution. For the analysis of protein secondary structure, we used IR-SSE analytical software (Jasco Co., Tokyo, Japan) in which the calibration curve data was prepared as a standard data file by multicomponent analysis (Partial Least Squares quantification model) based on the secondary-structural data of 17 proteins. In this program, the amide I band can be deconvoluted into four major bands: α-helix (1650-55 cm^{-1}), β-sheet (1625-40 cm^{-1}), β-turn (1655-75 cm^{-1}), and other conformation (1645-50 cm^{-1}). Proportions of secondary structures were obtained based on peak intensities at those amide-I bands. The mid-IR spectra for cellulose fiber were measured using BL6B synchrotron-radiation based infrared microspectroscopy at UVSOR. Samples were added on metal-coating plate and the measurement was performed by reflection mode with 64 scans.

8. Scanning-Electron Microscopy

We used an FE-SEM Supra40 scanning electron microscope (Carl Zeiss). After the lysozyme fibril was added on a glass slide and dried under atmosphere, the slide base was fixed to a sample holder by using conductive copper tape. The surface of the sample was observed using an acceleration voltage of 5.0 kV.

9. Congo-Red Staining

The lysozyme fibril before or after the far-infrared radiation was added on a glass slide and dried under atmospheric conditions. Congo-red was dissolved in PBS to be 0.2 mM concentration, and this solution (10 μL) was added to the amyloid sample on a slide.

The solution was dried at room temperature, and the surface was observed by using a polarized light microscope MVX 10 (Olympus, Tokyo).

10. Optical Microscopy

The cellulose fiber was added on a glass-slide base and dried under atmosphere. The surface of the sample was observed using an Area PIII-FX microscope (SK-Electronics Co., LTD., Kyoto, Japan) with a high-magnification object lens (×200–2000). Images were obtained using a 12 million-pixel CCD camera under a halogen lamp where the sample surface images were saved using Perfect Viewer 7 imaging software (SK-Electronics Co., LTD., Kyoto, Japan).

11. Small-Angle X-ray Scattering

X-ray scattering experiment was performed using the beamline BL8S3 in Aichi Synchrotron Radiation Center (Aichi, Japan). A suspension containing lysozyme fibril was placed on a Teflon sheet (1mm in depth), and the sample was surrounded and encapsulated by Kapton tape made of polyimide film (TERAOKA SEISAKUSHO CO., LTD., Tokyo). The sample cell was set vertically against the X-ray direction. The X-ray wavelength was 0.15 nm and the length from the sample to the camera was 45 cm for measurements. The scattering patterns were recorded using R-AXIS imaging plate (Rigaku, Japan) at an exposure time 600 s.
